# Feasibility and predictive factors on the completion of docetaxel plus S‐1 adjuvant chemotherapy in pathological stage III gastric cancer

**DOI:** 10.1002/ags3.12840

**Published:** 2024-07-03

**Authors:** Masayoshi Terayama, Manabu Ohashi, Kensei Yamaguchi, Daisuke Takahari, Rie Makuuchi, Masaru Hayami, Satoshi Ida, Koshi Kumagai, Takeshi Sano, Souya Nunobe

**Affiliations:** ^1^ Department of Gastroenterological Surgery, Gastroenterological Center, Cancer Institute Hospital Japanese Foundation for Cancer Research Tokyo Japan; ^2^ Department of Gastroenterological Chemotherapy, Gastroenterological Center Cancer Institute Hospital, Japanese Foundation for Cancer Research Tokyo Japan

**Keywords:** adjuvant chemotherapy, advanced gastric cancer, docetaxel, intramuscular adipose content, skeletal muscle mass

## Abstract

**Background:**

The standard adjuvant chemotherapy regimen for stage III gastric cancer is docetaxel plus S‐1 (DS) based on the results of the START‐II trials. However, in clinical practice some patients could not continue this intensive doublet chemotherapy because of limited tolerability. This study aimed to assess the practical feasibility of DS and elucidate the predictive factors for the completion of adjuvant DS therapy.

**Methods:**

Data from consecutive patients who underwent radical gastrectomy between 2018 and 2021 and were diagnosed with histopathologically confirmed stage III gastric cancer were retrospectively collected. First, the completion rate and adverse effects of DS were assessed. Second, the association between DS incompletion and patient backgrounds including body weight, skeletal muscle index (SMI), and intramuscular adipose content (IMAC) were examined.

**Results:**

Of 87 patients, 59 patients (67.8%) completed DS and dose reduction was required in 18 patients (20.6%). Neutropenia of grade 3 or higher was the most common hematological toxicity observed (17.2%). The most frequent nonhematological toxicity of grade 3 or higher was fatigue (6.9%), followed by diarrhea (5.7%), nausea (4.5%), and anorexia (4.5%). In a multivariate analysis, low SMI (*p* = 0.005) and high IMAC (*p* = 0.004) were significant risk factors for DS incompletion.

**Conclusions:**

DS adjuvant chemotherapy after radical gastrectomy for pathological stage III gastric cancer is acceptable, even in clinical practice, with respect to completion and toxicity. Additionally, the body composition factors such as SMI and IMAC might be useful in predicting incompletion of DS. These findings will help us to preoperatively select patients for DS.

## Graphical abstract

DS adjuvant chemotherapy after radical gastrectomy for pathological stage III gastric cancer is acceptable, even in clinical practice, with respect to safety and toxicity. The preoperative assessment of body composition factors, such as SMI and IMAC, could be useful in selecting patients who could completely receive DS.

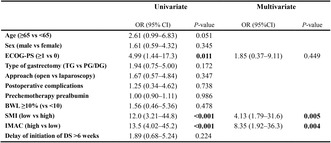

## 1 INTRODUCTION

Gastric cancer is the third leading cause of cancer deaths worldwide.^1^ Although the mainstay treatment for gastric cancer is surgery, advanced gastric cancer requires postoperative adjuvant chemotherapy to reduce the risk of recurrence and prolong survival after curative resection.^2^, ^3^ In Japan, the standard adjuvant chemotherapy regimen for pathological stage III (pStage III) gastric cancer is docetaxel plus S‐1 (DS) based on the results of the START‐II trials.^4^

The continuation of adjuvant chemotherapy is important to maximize curability. The feasibility of DS demonstrated by previous studies was derived from its good completion rate, at 71.0% in the START‐II trials^4^ and 77.4% in a phase II study reported by Fujitani et al.^5^ However, these advantageous results were obtained from strictly selected patients. In clinical practice, patients often show limited tolerability because of various health problems, and hence it is unclear whether to administer DS. Thus, our interest lies in the practical feasibility of DS when considering the chemotherapeutic indication, as well as how to continue DS and increase its completion rate to make DS more widely available.

The tolerance to chemotherapy differs for each patient.^6^ A previous study showed that the substantial variation and heterogeneity of body composition could greatly affect the feasibility of chemotherapy.^7^ In this regard, the skeletal muscle index (SMI) and intramuscular adipose content (IMAC) have been reported as useful parameters of body composition correlated with postoperative outcomes after gastrectomy.^8^, ^9^, ^10^ With adjuvant chemotherapy, low skeletal muscle mass was reported to be associated with the low relative dose intensity of adjuvant S‐1 chemotherapy after gastrectomy.^11^ Thus, measuring these parameters would be useful for selecting treatable patients if their values are associated with the continuation of DS.

In this study, to understand whether DS for postoperative patients with pStage III gastric cancer is feasible in daily practice and to determine predictive factors for the discontinuation of treatment, we evaluated the safety and toxicity of DS in patients treated at our hospital and investigated factors associated with the discontinuation of treatment. The results obtained from this study will provide us with valuable information regarding patients who cannot complete DS.

## 2 METHODS

### 2.1 Study design

The data of consecutive patients who were diagnosed with pStage III gastric cancer at the Japanese Foundation for Cancer Research between April 2018 and April 2021 were retrospectively collected. Among them, we selected patients who received DS chemotherapy and excluded patients for the following reasons: (1) procedures other than radical gastrectomy were performed; and (2) patients who received DS at another hospital. Tumor stages were classified according to the 14th edition of the Japanese Classification of Gastric Carcinoma (3rd English edition).^12^ This study was approved by the Institutional Review Board of the Cancer Institute Hospital (2023‐GB‐085).

### 2.2 DS therapy

Patients were given oral S‐1 twice daily for 2 consecutive weeks with intravenous docetaxel (40 mg/m^2^) on d 1, repeated every 3 weeks (one cycle). The initial dose of S‐1 was based on body surface area (BSA), as follows: BSA <1.25 m^2^, 80 mg/d; BSA ≥1.25 m^2^ and <1.5 m^2^, 100 mg/d; and BSA ≥1.5 m^2^, 120 mg/d. Treatment was started within 42 d after surgery. During the first course, patients were treated with S‐1 on d 1–14 of a 3‐week cycle. During the second to seventh courses, patients received intravenous infusion of docetaxel (40 mg/m^2^ body surface area) on d 1 of each cycle and S‐1 on d 1–14 of a 3‐week cycle. After the eighth course, S‐1 monotherapy (4 weeks, followed by 2 weeks of rest) was continued until 1 y after surgery. DS treatment was discontinued when the following criteria were observed: (1) recurrence of the underlying cancer; (2) requirement of more than a two‐level dose reduction of S‐1 or docetaxel; and (3) inability to receive S‐1. The doses of S‐1 and docetaxel were reduced in the event of grade 3 neutropenia, serum creatinine >1.5 mg/dL, other drug‐related nonhematological toxicities of grade 2 or higher, or any need for treatment suspension due to adverse effects.

### 2.3 Outcomes

We mainly analyzed practical outcomes of two items. First, the completion rate of seven courses and adverse events of DS were assessed. Second, the association between DS incompletion and patients' backgrounds including body compositions trends such as body weight (BW), skeletal muscle mass (SMM), and IMAC were analyzed. The adverse effects were defined by the most severe classification that occurred during the chemotherapy according to the Common Terminology Criteria for Adverse Events (CTCAE) v. 5.0. Poor compliance was defined as discontinuation of DS due to adverse events, the patient's refusal to continue chemotherapy, or recurrence. Perioperative clinicopathological data were evaluated, including age, sex, preoperative performance status (PS), surgical procedure, approach, prechemotherapy serum albumin, prealbumin and hemoglobin levels, creatinine clearance, and duration from surgery to the initiation of S‐1 administration. Then the association of these factors with poor compliance of DS was investigated. Postoperative complications were classified according to the Clavien–Dindo classification, and grade III or higher complications were analyzed. BW loss (BWL) was calculated by (preoperative BW–BW before chemotherapy)/preoperative BW. SMM was measured on preoperative computed tomography (CT) scans at the level of the third lumbar vertebra, having a density of −29 to 100 Hounsfield units, with a SYNAPSE VINCENT Volume Analyzer (Fujifilm Medical Co., Tokyo, Japan). IMAC was also calculated by preoperative CT scans at the umbilical level, having a density of −150 to −50 Hounsfield units. The SMI was calculated as follows: SMI = cross‐sectional area of the skeletal muscle (cm^2^)/height (m^2^). The cutoff value for SMI was set as 43.2 cm^2^/m^2^ for men and 34.6 cm^2^/m^2^ for women^13^ based on the Japanese population. The cutoff value for IMAC was set as −0.430 for men and −0.310 for women.^14^ Overall survival (OS) was calculated from the day of surgery to the day of death or last follow‐up. Recurrence‐free survival (RFS) was calculated from the day of surgery to the day of recurrence or death or last follow‐up.

### 2.4 Statistical analysis

Categorical and continuous variables were analyzed using the χ^2^ test and the Mann–Whitney test, respectively. Univariate and multivariate analyses were performed using logistic regression models. All variables with *p*‐values of <0.05 in the univariate analysis were included in the multivariate models. Statistical significance was defined as *p* < 0.05. All statistical analyses were performed using EZR (Saitama Medical Center, Jichi Medical University, Saitama, Japan), a graphical user interface for R (The R Foundation for Statistical Computing, Vienna, Austria).

## 3 RESULTS

### 3.1 Patient characteristics

**FIGURE 1:**
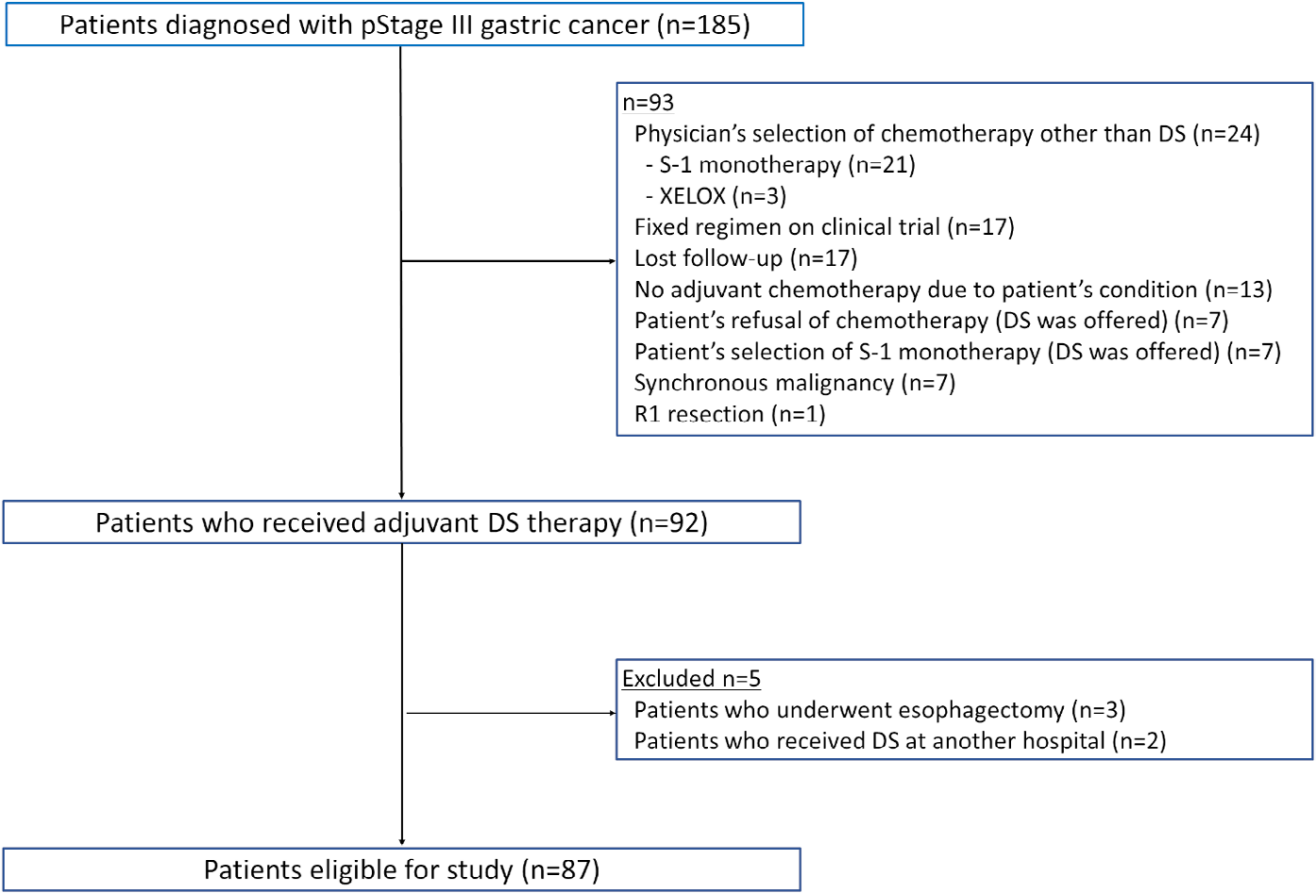
Patient selection. A total of 185 patients were diagnosed as having pStage III gastric cancer. Among them, 92 patients received adjuvant DS chemotherapy. After the exclusion of three patients who underwent esophagectomy and two patients who received DS chemotherapy at another hospital, 87 patients were eligible for this study. DS, docetaxel plus S‐1.

Patient selection is shown in Figure 1. A total of 185 patients were diagnosed as having pStage III gastric cancer. Among them, 92 patients received adjuvant DS chemotherapy. After exclusion, the data of 87 patients were finally collected in this study. Although 29 (33.3%) out of 87 patients did not meet the eligibility criteria in the START‐II trial,^4^ they received DS chemotherapy in clinical practice: 24 patients could not start chemotherapy within 42 d after surgery, two patients had anemia less than 9.0 g/dL at hemoglobin levels, and three patients could not undergo D2 lymphadenectomy because their preoperative diagnosis was early gastric cancer. Patient characteristics are listed in Table 1. The median age was 61 y (range, 50–79 y). Fifty men and 37 women were included. The numbers of patients with stage IIIA, IIIB, and IIIC disease were 39 (44.8%), 27 (31.0%), and 21 (24.1%), respectively. Three patients (3.4%) underwent proximal gastrectomy because their preoperative clinical diagnosis was early gastric cancer without lymph node metastasis. A laparoscopic approach was used in 29.9% of all patients.

**TABLE 1:** Patient characteristics.

Variables	All patients (*n* = 87)
Age*	61 (50–79)
Sex	
Male	50 (57.4)
Female	37 (42.5)
pStage	
IIIA	39 (44.8)
IIIB	27 (31.0)
IIIC	21 (24.1)
Tumor status	
T1b	1 (1.1)
T2	3 (3.4)
T3	29 (33.3)
T4a	48 (55.1)
T4b	6 (6.9)
Nodal status	
N0	2 (2.3)
N1	18 (20.6)
N2	35 (40.2)
N3a	24 (27.6)
N3b	8 (9.2)
ECOG‐PS	
0	72 (82.8)
1	15 (17.2)
Type of gastrectomy	
Total gastrectomy	39 (44.8)
Proximal gastrectomy	3 (3.4)
Distal gastrectomy	45 (51.7)
Approach	
Laparoscopy	26 (29.9)
Open	61 (70.1)

*Note*: Values in parentheses are percentages unless indicated otherwise; values are *median (range).

Abbreviation: ECOG‐PS, Eastern Cooperative Oncology Group–Performance Status.

### 3.2 Completion rate

Table 2 shows the completion rate of DS therapy in this study. Of 87 patients, 59 patients (67.8%) completed seven courses of docetaxel and 69 patients (79.3%) completed at least S‐1 treatment for 1 y except for the completion of DS therapy. Dose reduction of docetaxel was required in 18 patients (20.6%). In contrast, dose reduction of S‐1 was required in 46 patients (52.8%). The mean relative dose intensity of docetaxel and S‐1 was 74.8% and 79.7%, respectively. Among the 28 patients who discontinued DS, the reasons were as follows: 21 patients due to adverse events (75%), four due to recurrence (14%), and three due to patient refusal to continue (11%). Adverse events included hematological toxicities, such as primarily neutropenia and renal dysfunction, and nonhematological toxicities, which were mainly anorexia and diarrhea.

**TABLE 2:** Compliance with DS adjuvant chemotherapy.

Variable	Patients (*n* = 87)
Compliance of docetaxel + S‐1 and subsequent S‐1 monotherapy, *n* (%)	52 (59.7)
Treatment compliance of docetaxel, cycle number, *n* (%)	
0	2 (2.3)
1	8 (9.2)
2	2 (2.3)
3	11 (12.6)
4	0
5	5 (5.7)
6	59 (67.8)
Full‐dose completion, *n* (%)	36 (41.3)
Dose reduction, *n* (%)	18 (20.6)
Relative dose intensity (%)^a^	74.8 (68.2–81.3)
Treatment compliance of S‐1, postoperative month, *n* (%)	
3	7 (8.0)
6	5 (5.7)
9	6 (6.9)
12	69 (79.3)
Dose reduction, *n* (%)	46 (52.8)
Relative dose intensity (%)^a^	79.7 (74.5–84.9)

Abbreviation: DS, docetaxel plus S‐1.

^a^ Values are shown as mean with 95% confidence interval.

### 3.3 Adverse events

Table 3 shows the adverse events observed in patients who were administered DS. Neutropenia of grade 3 or higher was the most common hematological toxicity and was observed in 15 patients (17.2%). The most common nonhematological toxicity of grade 3 or higher was fatigue (6.9%), followed by diarrhea (5.7%), nausea (4.5%), and anorexia (4.5%). No significant differences were observed in the incidences of each adverse event between the complete and incomplete groups.

**TABLE 3:** Adverse events of Grade ≥3.

Adverse events	All (*n* = 87)	Complete (*n* = 59)	Incomplete (*n* = 28)	*p* Value
Neutropenia	15 (17.2)	8 (13.5)	7 (25.0)	0.229
Leukopenia	12 (13.7)	6 (10.1)	6 (21.4)	0.189
Anemia	5 (5.7)	2 (3.4)	3 (10.7)	0.323
Nausea	4 (4.5)	3 (5.1)	1 (3.6)	1
Diarrhea	5 (5.7)	2 (3.4)	3 (10.7)	0.323
Fatigue	6 (6.9)	5 (8.5)	1 (3.6)	0.659
Oral mucositis	2 (2.2)	1 (1.7)	1 (3.6)	0.543
Anorexia	4 (4.5)	1 (1.7)	3 (10.7)	0.096
Constipation	1 (1.1)	1 (1.7)	0	1
Dysgeusia	2 (2.2)	0	2 (7.2)	0.101
Hand–foot syndrome	1 (1.1)	1 (1.7)	0	1
Neuropathy	0	0	0	1
Rash	1 (1.1)	1 (1.7)	0	1
Hair loss	0	0	0	1
Edema	0	0	0	1
Pigmentation	0	0	0	1
Enteritis	1 (1.1)	1	0	1
Dizziness	0	0	0	1

### 3.4 Clinicopathological factors associated with the completion of DS therapy

Table 4 shows the associations between the completion of seven courses of DS therapy and various clinicopathological factors, excluding four patients with recurrence during DS therapy. Age (*p* = 0.028), poor PS (*p* = 0.034), low SMI (*p* < 0.001), and high IMAC (*p* < 0.001) were significantly associated with incomplete DS therapy. There were no significant differences observed in pathological tumor stage, T factor, and N factor. Total gastrectomy and a BWL of 10% or higher before chemotherapy were not correlated with DS incompletion (*p* = 0.23 and *p* = 0.533, respectively). Similarly, delayed initiation of DS did not affect DS completion (*p* = 0.295). Nutritional parameters, such as albumin and prealbumin, and renal function showed no relationship with DS incompletion.

**TABLE 4:** Association between the completion of DS therapy and clinicopathological factors.

Variable	Complete (*n* = 59)	Incomplete (*n* = 24)	*p‐*Value
Age*	59 (26–79)	69 (33–78)	0.028
Sex			
Male	33 (55.9)	17 (70.9)	0.229
Female	26 (44.1)	7 (29.1)	
ECOG‐PS			
0	54 (91.5)	17 (71.9)	0.034
1	5 (8.5)	7 (29.1)	
Surgical procedure			
Total gastrectomy	23 (38.9)	13 (54.2)	0.23
Proximal or distal gastrectomy	36 (61.1)	11 (45.8)	
Approach			
Laparoscopic	20 (33.9)	6 (25.0)	0.602
Open	39 (56.1)	18 (75.0)	
Postoperative complications			0.737
Clavien–Dindo grade ≥3	8 (13.6)	4 (16.6)	
pStage			0.898
IIIA	27 (45.8)	12 (50.0)	
IIIB	17 (28.8)	8 (33.3)	
IIIC	15 (23.7)	4 (16.7)	
Tumor status			0.899
T1b	1 (1.7)	0	
T2	3 (5.1)	0	
T3	20 (33.9)	8 (33.3)	
T4a	31 (52.5)	14 (58.3)	
T4b	4 (6.8)	2 (7.1)	
Nodal status			0.484
N0	1 (1.7)	1 (4.2)	
N1	10 (16.9)	8 (33.3)	
N2	25 (42.4)	9 (37.5)	
N3a	18 (30.5)	4 (16.6)	
N3b	5 (8.5)	2 (8.3)	
Prechemotherapy serum albumin			1
>3.5	56 (94.9)	23 (95.9)	
≤3.5	3 (5.1)	1 (4.1)	
Prechemotherapy prealbumin *	20.3 (17.6–23.9)	20.5 (10.4–29.0)	0.873
Creatinine clearance *	95.7 (76.4–115.4)	74.4 (38.3–161.8)	0.073
BWL before chemotherapy			
<10%	50 (84.7)	19 (79.2)	0.533
≥10%	9 (15.3)	5 (20.8)	
Skeletal muscle index			
≥Cut off score	36 (61.0)	3 (12.5)	<0.001
<Cut off score	23 (39.0)	21 (87.5)	
Intramuscular adipose content			
≥Cut off score	6 (10.1)	10 (41.7)	<0.001
<Cut off score	53 (89.9)	14 (58.3)	
Initiation of DS			0.295
≤6 weeks	44 (74.5)	15 (62.5)	
>6 weeks	15 (25.4)	9 (37.5)	

*Note*: Values in parentheses are percentages unless indicated otherwise; values are *median (range).

Abbreviations: BWL, body weight loss; DS, docetaxel plus S‐1; ECOG‐PS, Eastern Cooperative Oncology Group–Performance Status.

### 3.5 Predictive factors of the completion with DS therapy

In the multivariate analysis excluding four patients with recurrence during DS therapy, low SMI (*p* = 0.005) and high IMAC (*p* = 0.004) were significant risk factors for DS incompletion (Table 5).

**TABLE 5:** Multivariate analysis of incompletion of DS therapy.

	Univariate		Multivariate	
	OR (95% CI)	*p*‐Value	OR (95% CI)	*p*‐Value
Age (≥65 vs. <65)	2.61 (0.99–6.83)	0.051		
Sex (male vs. female)	1.61 (0.59–4.32)	0.345		
ECOG‐PS (≥1 vs. 0)	4.99 (1.44–17.3)	0.011	1.85 (0.37–9.11)	0.449
Type of gastrectomy (TG vs. PG/DG)	1.94 (0.75–5.00)	0.172		
Approach (open vs. laparoscopy)	1.67 (0.57–4.84)	0.347		
Postoperative complications	1.25 (0.34–4.62)	0.738		
Prechemotherapy prealbumin	1.00 (0.90–1.11)	0.986		
BWL ≥ 10% (vs. <10)	1.56 (0.46–5.36)	0.478		
SMI (low vs. high)	12.0 (3.21–44.8)	<0.001	4.13 (1.79–31.6)	0.005
IMAC (high vs. low)	13.5 (4.02–45.2)	<0.001	8.35 (1.92–36.3)	0.004
Delay of initiation of DS >6 weeks	1.89 (0.68–5.24)	0.224		

Abbreviations: BWL, body weight loss; DG, distal gastrectomy; DS, docetaxel plus S‐1; ECOG‐PS, Eastern Cooperative Oncology Group–Performance Status; IMAC, intramuscular adipose content; PG, proximal gastrectomy; SMI, skeletal muscle index; TG, total ga6strectomy.

### 3.6 Survival and recurrence

The patients in the complete group demonstrated better 3‐y OS and 3‐y RFS rates than those in the incomplete group (Figure S1). Table 6 summarizes recurrence sites. Forty‐three patients (49.4%) experienced recurrence: 21 patients (35.6%) in the complete group and 22 patients (78.5%) in the incomplete group. In detail, the most frequent recurrence site was the peritoneum (31.0%) and significantly more patients in the incomplete group experienced peritoneal metastasis than those in the complete group. The time to recurrence analysis showed postoperative recurrence more frequently occurred in the incomplete group than the complete group (Figure S2).

**TABLE 6:** Recurrence site.

Site	All (*n* = 87)	Complete (*n* = 59)	Incomplete (*n* = 28)	*p*‐Value
Peritoneum	27 (31.0)	11 (18.6)	16 (57.1)	<0.001
Liver	6 (7.2)	4 (6.8)	2 (7.1)	1
Lymph nodes	7 (8.4)	4 (6.8)	3 (10.7)	0.676
Bone	2 (2.4)	1 (1.7)	1 (3.6)	0.543
Lung	1 (1.2)	1 (1.7)	0	1
Total	43 (49.4)	21 (35.6)	22 (78.5)	<0.001

## 4 DISCUSSION

This study aimed to assess the practical feasibility of DS and identify factors that significantly affected the compliance with DS in pStage III gastric cancer. The completion of DS and its dose reduction rate were acceptable at 67.8% and 20.6%, respectively. In the multivariate analysis, poor PS (PS = 1) and the deterioration of preoperative body composition, such as low SMI and high IMAC, were significantly associated with the incompletion of DS. In contrast, older age (≥65 y), BWL before chemotherapy, and total gastrectomy were not correlated with this. Our results demonstrated that adjuvant DS was a feasible treatment for patients with pStage III gastric cancer in clinical practice, although it required some attention to those who had a poor PS and deterioration of SMI and IMAC. These findings will help us to preoperatively select patients who can complete DS adjuvant chemotherapy.

The completion rate of DS was 67.8% in our study, which was comparable to previous studies. The low dose reduction rate at 20.6% might be related to this favorable result compared with 28% in the START‐II trial (4) and 56% in the other phase II study (5). A major reason was the good pharmacokinetics of docetaxel in our study population. A well‐known hematological toxicity of docetaxel is myelosuppression.^15^, ^16^ In our patients, the incidence of grade 3 or 4 neutropenia was 17.2%, which was less frequent than in a previous study (28%), despite 36 out of 59 patients with DS completion (61.0%) being administered full‐dose DS therapy. Older age is also reported to be a risk factor of hematological toxicity^17^; however, in our population relatively large numbers of younger patients (median age, 61 y) were collected, which might be related to the low incidence of hematological toxicities, and subsequently led to the low rate of dose reduction. Moreover, the patients in our study showed good renal function. The renal dysfunction represented by low creatinine clearance largely affected the discontinuation of chemotherapy.^18^ Thus, high serum creatinine clearance at 81.8 mg/dL, even in the incomplete group, might have contributed to the low rate of dose reduction. Overall, although this was real‐world data, patients were carefully selected due to the early initiation of DS therapy, which might have led to these favorable outcomes.

Our study focused on preoperative body composition and suggested that low SMI and high IMAC had an impact on the poor compliance with DS. Furthermore, the incompletion of DS therapy was associated with poor OS and RFS, which indicated the significance of predicting the completion of DS therapy. There have been few studies of quantitative targets for predicting tolerability to docetaxel exposure. Body surface area is conventionally used for decisions regarding drug dose; however, it is affected by body composition changes. In this regard, our results demonstrated the great predictive values of SMI and IMAC in determining those who could not complete seven courses of DS. The mechanism underlying the association between low skeletal muscle quality or quantity and the discontinuation of chemotherapy remains unclear. Skeletal muscle loss, as a representative parameter of skeletal muscle quantity, is related to the reduced homeostatic reserves observed in malnutrition, immune system impairment, and organ failure.^19^, ^20^ Similarly, IMAC as a representative parameter of skeletal muscle quality, whose increase indicates the infiltration of fat tissue, was accompanied by skeletal muscle loss. The European Working Group on Sarcopenia in Older People recommended the measurement of reduced skeletal muscle quality (cf. IMAC) in addition to reduced quantity (cf. SMI) to evaluate sarcopenia in its guidelines published in 2018.^21^ In patients undergoing gastrectomy, the postoperative loss of skeletal muscle quality or quantity is especially accelerated because of inadequate protein intake and decreased physical activity, resulting in an estimated 3.8%–6.2% loss of skeletal muscle after gastrectomy.^22^, ^23^ Thus, preoperative skeletal muscle reserves are important. As for evaluation tools, although body surface area has been used to calculate the appropriate dose of chemotherapy, both SMI and IMAC calculated by CT scans are reliable and available indicators that are preoperatively available.

To improve the completion rate of DS, we should consider preoperative interventions and appropriate dose inductions for patients with low SMI or high IMAC. Nutritional support and exercise can be effective interventions for individuals with muscle loss. Adequate calorie and protein intake are important for muscle synthesis and repair, which can help improve skeletal muscle index (SMI) and reduce intramuscular adipose tissue (IMAC). Additionally, regular physical activity, particularly resistance training, can increase muscle mass and strength, further improving SMI and reducing IMAC. A pilot study conducted by Yamamoto et al showed that preoperative muscle training and protein intake for more than 3 weeks resulted in a meaningful increase in skeletal muscle mass.^24^ Although some patients could not receive this support because of their physical vulnerability and malignant condition, we could improve the patients' sarcopenic status through preoperative exercise and nutritional support. Another considerable intervention is drug dose adjustment to prevent adverse effects. From our data, the main reason for DS discontinuation was adverse effects. A previous study reported that sarcopenia may influence chemotherapy pharmacokinetics, which could be correlated with the adverse effects of chemotherapy in several cancer types.^25^, ^26^ Traditionally, the dose of chemotherapy is generally dependent on the patient's body surface area, and changes in body composition may not be considered. However, previous reports implied that changes in body composition, such as less muscle or greater adiposity, could alter the distribution of chemotherapy agents and consequently increase the toxic effects.^27^ Hence, some sarcopenic patients who are treated based on body surface area tend to receive more chemotherapeutic agent than originally required (with a relatively low body composition). This might be one of the reasons why sarcopenic patients receiving chemotherapy experienced chemotherapeutic toxicities, resulting in early withdrawal and poor survival outcomes.^28^, ^29^, ^30^ Considering these situations, we proposed that adjustment to an appropriate dose intensity according to low SMI or IMAC was desirable when initiating adjuvant DS chemotherapy.

This study has several limitations. First, this was a single‐institutional retrospective study with a small sample size. Second, only ~10% of recruited patients were followed up after gastrectomy due to personal reasons, with some returning to their country of origin or facing difficulties in attending outpatient clinics because of the COVID‐19 pandemic. These losses may have affected our results. Third, the SMI and IMAC cutoff values might differ depending on the patient's background, such as ethnicity. Fourth, the correlation between postoperative SMI/IMAC and the completion of DS could not be investigated because CT examination was scarcely performed from the date of surgery to the initiation of chemotherapy. In this study, cutoff values were derived from a previous study based on Japanese populations. Thus, different populations might have different cutoff values. Finally, in contrast to the previous study with S‐1,^31^ the present study did not find BWL to be a predictor of DS therapy continuation. One possible reason for this discrepancy lies in the difference in cutoff values for BWL used in the studies. While the previous studies set the cutoff at 15%, we chose a lower threshold of 10% because only three patients (3.4%) experienced BWL of 15% or higher. Including patients with less severe BWL in our study may have diluted the potential impact of BWL on therapy continuation.

In conclusion, DS adjuvant chemotherapy is acceptable, even in clinical practice, with respect to completion and toxicity. Additionally, the assessment of preoperative body composition such as SMI and IMAC, rather than BW, might be useful for predicting the incompletion of DS therapy after radical gastrectomy for pathological stage III gastric cancer. Physicians should pay careful attention to preoperative SMI or IMAC and may consider some interventions to not only improve DS compliance but also to improve survival outcomes.

## AUTHOR CONTRIBUTIONS

Conceptualization, Data curation & Formal analysis: MT and MO; Investigation: MT, Methodology: MT and MO, Software: MT; Supervision, Validation & Visualization: MT and MO, Writing – original drafts: MT and MO. Writing –review & editing: MT, MO, KY, DT, RM, MH, SI, KK, TS, and SN.

## FUNDING INFORMATION

None.

## CONFLICT OF INTEREST STATEMENT

The authors declare that they have no conflicts of interest.

## ETHICAL STANDARDS

All procedures followed were in accordance with the ethical standards of the responsible committee on human experimentation (institutional and national) and with the Declaration of Helsinki of 1964 and its later versions. Informed consent to be included in the study, or the equivalent, was obtained from all patients.

Approval of the research protocol: N/A.

Informed Consent: N/A.

Registry and the registration no. of the study/trial: N/A.

Animal studies: N/A.

## Supporting information


Figure S1

Figure S2

## Data Availability

The datasets generated and/or analyzed during the current study are available from the corresponding author on reasonable request.
