# Supplementary figures and images for: Feasibility and predictive factors on the completion of docetaxel plus S‐1 adjuvant chemotherapy in pathological stage III gastric cancer
Source: Ann Gastroenterol Surg. 2024 Jul 3;9(1):60–8. doi: 10.1002/ags3.12840 (PMC11693541; doi:10.1002/ags3.12840)

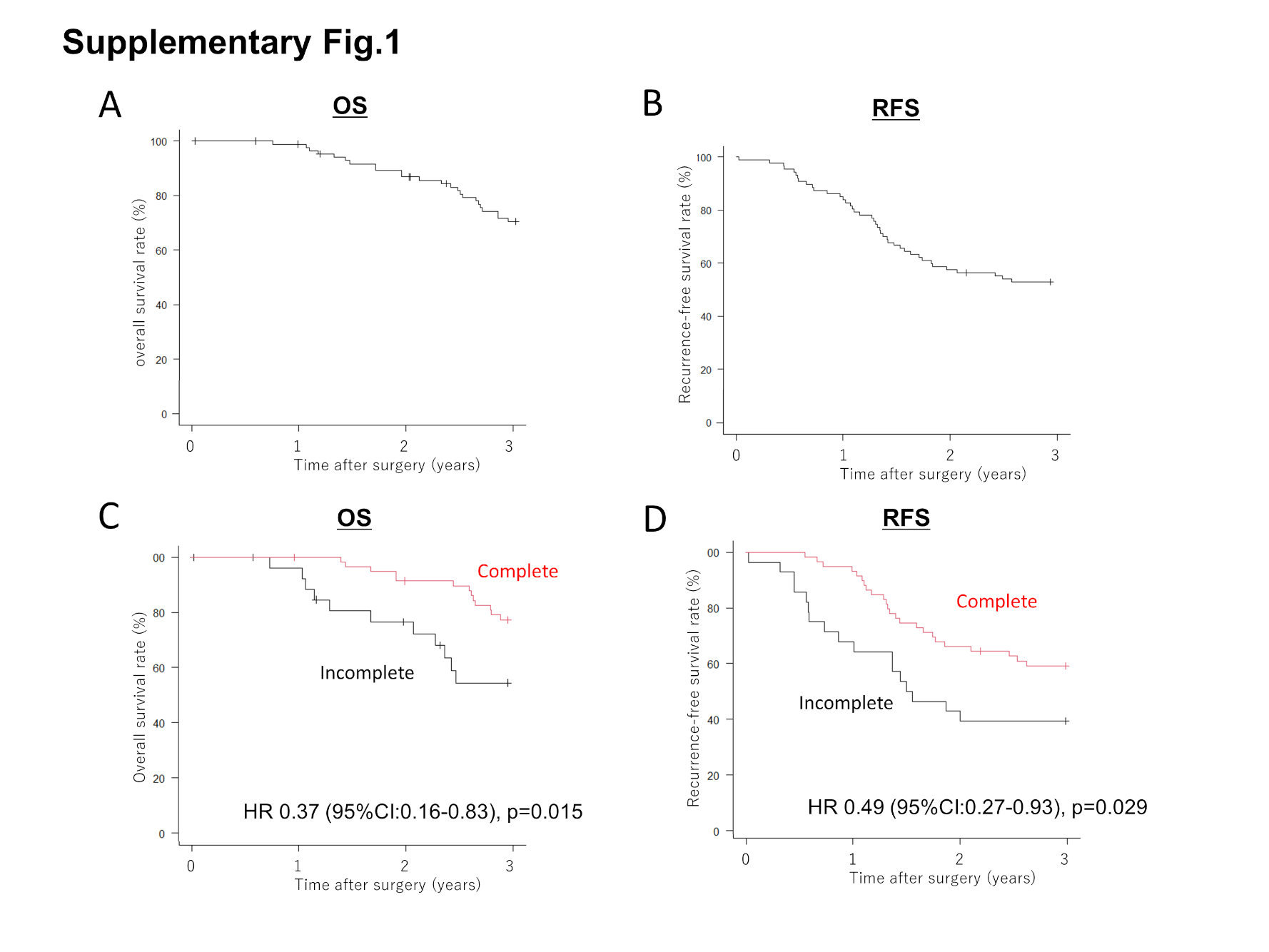

Supplement: Supplementary file 1 — Figure S1 [file AGS3-9-60-s002.tif]

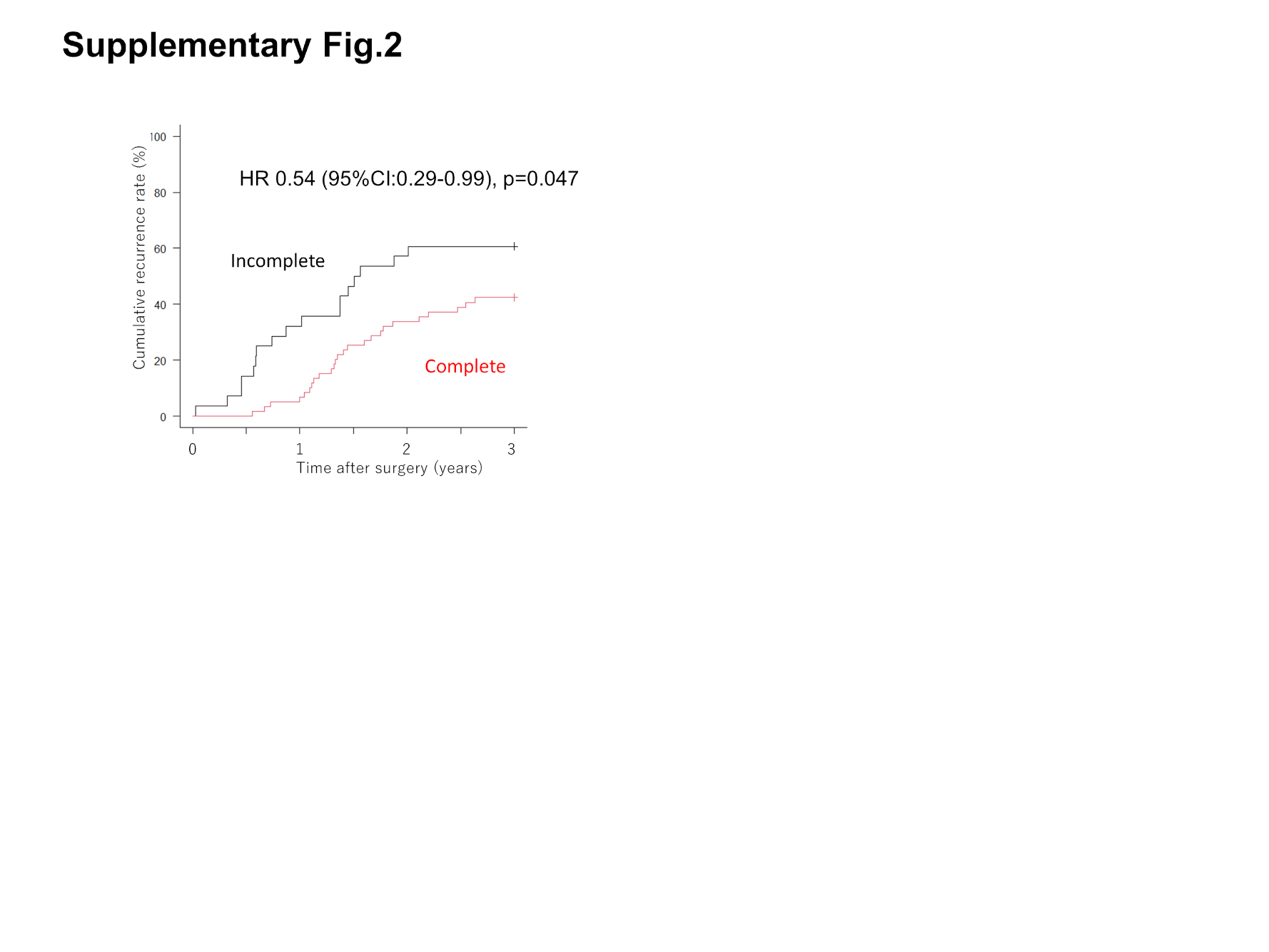

Supplement: Supplementary file 2 — Figure S2 [file AGS3-9-60-s001.tif]
